# Comprehensive insights into herbicide resistance mechanisms in weeds: a synergistic integration of transcriptomic and metabolomic analyses

**DOI:** 10.3389/fpls.2023.1280118

**Published:** 2023-10-11

**Authors:** Madhab Kumar Sen, Soham Bhattacharya, Rohit Bharati, Katerina Hamouzová, Josef Soukup

**Affiliations:** ^1^ Department of Agroecology and Crop Production, Faculty of Agrobiology, Food and Natural Resources, Czech University of Life Sciences Prague, Suchdol, Czechia; ^2^ Department of Crop Sciences and Agroforestry, Faculty of Tropical AgriSciences, Czech University of Life Sciences Prague, Suchdol, Czechia

**Keywords:** abiotic stresses, herbicide resistance, metabolomics, omics technologies, transcriptomic regulation

## Abstract

Omics techniques, including genomics, transcriptomics, proteomics, and metabolomics have smoothed the researcher’s ability to generate hypotheses and discover various agronomically relevant functions and mechanisms, as well as their implications and associations. With a significant increase in the number of cases with resistance to multiple herbicide modes of action, studies on herbicide resistance are currently one of the predominant areas of research within the field of weed science. High-throughput technologies have already started revolutionizing the current molecular weed biology studies. The evolution of herbicide resistance in weeds (particularly via non-target site resistance mechanism) is a perfect example of a complex, multi-pathway integration-induced response. To date, functional genomics, including transcriptomic and metabolomic studies have been used separately in herbicide resistance research, however there is a substantial lack of integrated approach. Hence, despite the ability of omics technologies to provide significant insights into the molecular functioning of weeds, using a single omics can sometimes be misleading. This mini-review will aim to discuss the current progress of transcriptome-based and metabolome-based approaches in herbicide resistance research, along with their systematic integration.

## 1 Introduction

Herbicide resistance among weeds has become a major reason for food security problems since the early years of synthetic herbicide development. Since then, there has been a continuous increase in the frequency of herbicide resistance cases, a major plant protection challenge encountered by farmers across the planet. Herbicides have been used to control weeds and increase the quality and quantity of major crops since their discovery. However, repeated use of similar herbicidal modes of action (MOAs) and the associated selection pressure have led to the evolution of herbicide resistance in varieties of economically important weedy species such as *Alopecurus myosuroides* (Lan et al., 2022; Goldberg-Cavalleri et al., 2023), *Amaranthus palmeri* (Manicardi et al., 2023), *Apera spica venti* (Košnarová et al., 2021; Papapanagiotou et al., 2022), *Bromus sterilis* (Sen et al., 2021), *Lolium* spp (Tehranchian et al., 2019; Ma et al., 2020; Zhu et al., 2023). Recent studies showed that the majority of these weeds were also developing multiple resistances against herbicides with more than one mode of action (Tehranchian et al., 2019; Ma et al., 2020; Košnarová et al., 2021; Lan et al., 2022; Papapanagiotou et al., 2022; Zhu et al., 2023). From a molecular point of view, herbicide resistance mechanisms might be categorized into target-site based resistance (TSR) and non-target-site based resistance (NTSR) (Torra and Alcántara-de la Cruz, 2022). Briefly, TSR comprises single or multiple nucleotide polymorphisms (point mutations), codon deletion (till date there is only one evidence in the case of protoporphyrinogen oxidase in *Amaranthus* sp.) and target gene overexpression (either via transcriptional regulatory mechanisms or via gene amplification) (Gaines et al., 2020). On the other hand, NTSR constitutes enhanced metabolism [via cytochrome P450s (Cyp450s), glutathione S-transferases (GSTs) and glycosyltransferases (GTs)], reduced uptake and translocation, vacuolar sequestration and increased ability to deal with oxygen radicals (Gaines et al., 2020). More details on these mechanisms can be found in Gaines et al., 2020. Reports show that the NTSR is more complicated and trickier to understand than the TSR (Jugulam and Shyam, 2019). Nevertheless, irrespective of the specific resistance mechanism, herbicide-resistant weeds, if left uncontrolled, will pose a significant threat to the global agricultural sector.

High-throughput molecular technologies (such as genomics, proteomics, metabolomics, transcriptomics etc.) have the potential to offer great analytical opportunities to understand and identify the mechanisms of herbicide resistance, thus leading to the identification of novel herbicidal MOAs (Ravet et al., 2018). Development of herbicide resistance in weeds is similar to that of abiotic stress resistance development in plants and can provide insights into the stress resistance mechanisms in plants in general (Hamouzová et al., 2023). Even though during the last decade, continuous progress has been made in the field of DNA, RNA, and protein-based omics studies in herbicide resistance, substantial research areas are yet to be explored. Additionally, to our knowledge, inadequate initiatives have been taken to integrate multiple omics-based studies to elucidate the mechanisms of herbicide resistance in economically significant weeds. Hence, in this mini-review, we will confine our discussion to transcriptomics, metabolomics and the need for their systematic integration to unravel the molecular mechanisms of herbicide resistance in weeds. The transcriptome and metabolome represent two key layers of biological information that can provide novel insights into how weeds develop resistance against herbicides. While transcriptomics provides a global overview on the expression profile of the genes, metabolomics provides a global overview on metabolic pathways and their regulation (Maroli et al., 2018a).

## 2 Weeds’ response to herbicide stress: effects of herbicide stress on weeds’ metabolic pathways and relevant genes involved

Among the metabolic pathways, the most important ones are carbohydrate metabolism, amino acid metabolism, polyamine metabolism, and lipid metabolism (Arbona et al., 2013). Carbohydrate metabolism is directly linked to photosynthetic performance and is the primary source of energy during stressful periods. Photosystem inhibitors such as atrazine, chlorotoluron and metribuzin are known to be involved in the disruption of the light-capturing reactions and electron transport chain in chloroplasts. These herbicides inhibit the production of ATP and NADPH (via competitive binding to the plastoquinone binding site (QB) on the D1 protein, which is encoded by the *PsbA* gene), which eventually affects carbohydrate metabolism in the weeds (Ma et al., 2020; Košnarová et al., 2021; Yang et al., 2022a). As of 2022 (according to https://www.weedscience.org/Home.aspx, accessed on July 23^rd^, 2023), 87 cases of resistance against PSII-inhibiting herbicides have been reported globally. Mutations in the *PsbA* gene and increased target gene and/or protein expression were identified as the mechanisms of resistance (Košnarová et al., 2021; Yang et al., 2022b). Besides interfering with the electron transport chain, herbicides can also have adverse effects on starch accumulation, sucrose transport and plant’s respiration process, which eventually will affect carbohydrate metabolism pathways. In addition to these, accumulation of sugars with no energetic roles (such as oligosaccharides) has also been detected in several plant species, as a response to abiotic stresses. These compounds are well-known for their indirect associations with reactive oxygen species (ROS) scavenging (Arbona et al., 2013). ROS scavenging is a vital mechanism while preventing oxidative stress and associated cellular damage. In the context of herbicide resistance, metabolism of herbicides via metabolic enzymes leads to oxidative stress within the weed. In answer to this, resistant weeds activate enzymes such as superoxide dismutase, catalase, and peroxidases and perform ROS scavenging, which eventually provides them survival advantages over their susceptible counterparts. However, very few such research on herbicide resistant-weeds have been conducted till date. Apart from carbohydrate metabolism, amino acid and lipid metabolism are also significant metabolic pathways. Acetolactate synthase and 5-enolpyruvylshikimate-3-phosphate synthase are among the most important enzymes involved in amino acid metabolic pathways and targeted by commercially important herbicides (Tan et al., 2006). Both of these enzymes are involved in the synthesis of aromatic amino acids (phenylalanine, tyrosine, and tryptophan). These amino acids play important roles during protein synthesis as well as in the production of various plant defense-related secondary metabolites. Among the important enzymes involved in fatty acid metabolism targeted by herbicides is Acetyl-CoA carboxylase (ACCase). ACCase plays a critical role in fatty acid synthesis (Takano et al., 2020). During stress periods, lipids play vital roles, which includes membrane fluidity and integrity, as signaling molecules and oxidative stress management (Liu et al., 2019).

## 3 Integrated transcriptomics-metabolomics: understanding the adaptation, tolerance, and resistance in plants

In the era of systems biology research, biological processes and gene regulatory networks can be dynamic and hard to interpret. Advanced omics technologies such as metabolomics and transcriptomics, have contributed significantly to explicating the molecular mechanisms of plant responses to different stresses. In the last few decades, information derived from next-generation sequencing technologies paired with advanced statistical and computational approaches has allowed for the characterization of genes involved in plant abiotic stress (Mehta et al., 2019; Sahoo et al., 2020; Aruna Kumara and Thiruchchelvan, 2021). Previously, thorough characterization of genes involved in stress responses was limited to crops and model systems because of the requisite genomic resources, but recent advancements permit the study of non-model species such as weeds. Despite challenges like the development of suitable methods and pipelines, RNA-seq transcriptome sequencing has been extensively used for the identification of gene families involved in herbicide resistance, particularly the NTSR (Zhao et al., 2017; Bai et al., 2020; Chen et al., 2021). Till date, there are many evidence of the use of transcriptomics and metabolomics in herbicide-resistance research. While transcriptomics can identify the differentially expressed genes, the metabolites are the ultimate result of controlled gene transcription and hence the metabolome can be considered the basis of the system’s observed phenotype. In the study conducted by Wrzesińska-Krupa et al., the authors used a *de novo* transcriptome of *Apera spica-venti* to perform RNA-sequencing analysis of pinoxaden-resistant and susceptible plants. They identified several important genes responsible for herbicide resistance, such as isoforms of *UDP-GT*s and *GST*s (along with others) as the prime candidate genes (Wrzesińska-Krupa et al., 2023). In another study, the authors used a comparative RNA-seq transcriptomic approach to understand how florpyrauxifen-benzyl treatment can affect phytohormone biosynthesis and signal transduction in resistant and susceptible *Echinochloa crus-galli* (L.) P. Beauv (Jin et al., 2023). Zhao et al., used transcriptome profiling and identified twenty-four contigs (including isoforms of *CytP450s*, *GSTs*, *GTs*, *ABC*-*transporters*, and others) involved in mesosulfuron-methyl resistance in *Alopecurus aequalis* (Zhao et al., 2017).

In addition to the RNA-seq studies, metabolomics has also been used in herbicide-resistance studies. The metabolome refers to the complete set of metabolites present within a biological sample. Even though the metabolome represents the smallest domain (among genome, transcriptome, proteome, and metabolome), it is more chemically complex and diverse than its counterparts (Kumar et al., 2017). In the context of understanding herbicide resistance in weeds, the metabolome can provide additional information since it represents additional levels of regulation as well as the end products of regulatory processes (Boonchaisri et al., 2020). For example, in the study conducted by Zulet-Gonzalez et al., the authors used non-targeted gas chromatography-mass spectrometry (GC-MS) and liquid chromatography–mass spectrometry (LC–MS) metabolomic profiling and examined the phytotoxic effects of glyphosate on sensitive and resistant populations of *Amaranthus palmeri* S. Wats. They didn’t find any differences in the metabolic profiles in the absence of herbicide treatment. However, upon treatment with glyphosate, they detected reduced concentrations of quercetin and its derivatives only in the resistant plants (Zulet-Gonzalez et al., 2023). In another study conducted by Tafoya-Razo et al., the authors conducted metabolic fingerprinting of susceptible and resistant common *Avena fatua* L. populations using Desorption Electrospray Ionization-Mass Spectrometry (DIESI-MS). The authors found that metabolomic fingerprinting can be successfully used to study the diversities of herbicide-resistance mechanisms, which can be used to study the “geographic mosaic of resistance” (Tafoya-Razo et al., 2022). From the perspective of NTSR mechanisms, metabolites can regulate the constitutive and induced expression of Cyp450s, GSTs, and GTs, thus leading to modulation of their activities during herbicide stress. In a different way, metabolites can also regulate the expression of the NTSR genes by altering the chromatin structure of the gene’s regulatory regions. To date, such studies are yet to be conducted. Comparative metabolomics among the treated and untreated resistant and susceptible plants will enable us to discover the detailed metabolites that are involved in herbicide detoxification, along with determining their relative abundance and activities. **Table 1** summarizes the list of transcriptomics and metabolomics-based research works related to herbicide sensitivities.

**Table 1:** Some important transcriptomics and metabolomics-related research works identified in major weedy species.

Approach	Herbicide active ingredients	Weed species	Short description	Reference
Transcriptomics	Clodinafop-propargyl	*Polypogon fugax*	The present study compared ACCase-resistant to ACCase-sensitive *P. fugax* by transcriptomics. The authors found differentially expressed unigenes related to ACCase-resistant in *P. fugax*.	Zhou et al., 2017
	Fenoxaprop-P-ethyl		Transcriptomic profiling identified 28 detoxifying enzyme genes which are herbicide-induced upregulated in the resistant biotype than the susceptible biotype.	Zhao et al., 2022a
	Cyhalofop-butyl	*Leptochloa chinensis*	Transcriptome analysis has been employed to identify candidate genes that may be involved in cyhalofop-butyl tolerance. This analysis identified three *cytochrome P450 genes* and three *ATP-binding cassette transporter* genes.	Chen et al., 2021
	2,4-D dimethylamine salt, dicamba diglycolamine salt, halauxifen-methyl	*Erigeron canadensis*	The study of transcriptomics used in *Erigeron canadensis* to auxin herbicide application revealed that auxin herbicide application enhanced the expression of the key abscisic acid biosynthetic gene which led to a rapid biosynthesis of abscisic acid (ABA) causing plant death.	McCauley et al., 2020
	Fenoxaprop-P-ethyl, chlorotoluron,	*Alopecurus myosuroides*	From the transcriptome data of the present study two AmGSTF1 variants were identified which were functionally linked to NTSR and enhanced herbicide metabolism.	Franco-Ortega et al., 2021
	Fenoxaprop, pendimethalin		Transcriptomics study has been used to investigate the evolution of MHR in populations of the weed blackgrass. The results found over 4500 genes showed perturbation in their expression in MHR versus herbicide-sensitive (HS) plants.	Tétard-Jones et al., 2018
	Florpyrauxifen-Benzyl	*Echinochloa crus-galli*	Comparative transcriptomic analysis of florpyrauxifen-benzyl treatment on phytohormone transduction between resistant and susceptible plants found a stronger auxin response and higher expression of related genes involved in ethylene and abscisic acid biosynthesis in S biotypes and signal transduction after herbicide treatment and also brassinolide receptor gene was upregulated and higher expressed in S biotype.	Jin et al., 2023
	Flucarbazone	*Avena fatua*	Transcriptome has been used to compare constitutive changes in multiple-herbicide resistance and herbicide-susceptible *Avena fatua* associated with non-target site resistance. The findings promote that intensive use of the herbicide has been selected for MHR populations with altered, constitutively regulated patterns of gene expression that are similar to abiotic stress-tolerant plants.	Keith et al., 2017
	Glufosinate	*Amaranthus palmeri*	Transcriptome analysis of *A. palmeri* plants have been done with differential tolerance to glufosinate herbicide. The results identified 567 differential expressed genes between sensitive and treated biotypes. 210 genes were highly induced in the treated T biotype than in the S biotype.	Salas-Perez et al., 2018
	Glyphosate	*Eleusine indica*	RNA-seq study was performed to investigate the glyphosate resistance mechanism in *Eleusine indica*. Research findings confirmed that two UniGenes (PFK, EPSPS) were strongly associated with target-site resistance, and two GST-annotated UniGenes may play a role in metabolic glyphosate resistance in goosegrass.	Chen et al., 2017
		*Conyza bonariensis*	The present RNA-Seq study was performed with the goal of identifying differentially expressed candidate genes related to non-target site glyphosate resistance in *C. bonariensis*. The present study revealed 41 new candidate NTSR genes in addition to two genes coding for antioxidant enzyme catalase, peroxidase, and superoxide dismutase.	Piasecki et al., 2019
	Mesosulfuron-Methyl	*Alopecurus aequalis*	This is the first large-scale transcriptome-sequencing study to identify NTSR genes in *A. aequalis* that uses the Illumina platform. This work demonstrates that NTSR is likely driven by the differences in the expression patterns of a set of genes.	Zhao et al., 2017
			Researchers identified four potential herbicide metabolism-related genes (*CYP709C56, CYP71R18, CYP94C117*, and *CYP94E14*) by transcriptional analysis which have higher expressions in the resistant plant.	Zhao et al., 2022b
		*Aegilops tauschii*	Transcriptomics has been used to investigate non-target-site resistance. The result showed that cytochrome P450s and GSTs involved in enhanced mesosulfuron-methyl metabolism in *A. tauschii*.	Zhang et al., 2022a
		*Beckmannia syzigachne*	Two ATP-binding cassette (ABC) transporter genes (*ABCB25* and *ABCC14*) were found upregulated in the R population by RNA-sequencing.	Wang et al., 2021
	Mesotrione	*Amaranthus tuberculatus*	RNA-sequence analysis indicated that the response of HPPD-herbicide against resistant and susceptible genotypes is rapid and established as soon as 3 hours after herbicide treatment.	Kohlhase et al., 2019
	Pinoxaden	*Apera spica-venti*	The results obtained from RNA-sequencing analysis showed the prime candidate genes responsible for herbicide resistance such as those encoding 3-ketoacyl-CoA synthase 12-like, UGT75K6, UGT75E2, UGT83A1-like, GSTU1, and GSTU6.	Wrzesińska-Krupa et al., 2023
	Tribenuron-methyl	*Myosoton aquaticum*	Transcriptome analysis was performed to identify candidate genes involved in the metabolic resistance of *Myosoton aquaticum*. Four genes *CYP734A1, CYP76C1, CYP86B1*, and *ABCC10* were identified which could play an essential role in the metabolic resistance of the NTSR mechanism.	Liu et al., 2018
	Trifloxysulfuron	*Poa annua*	The present transcriptomic study revealed differential gene expression associated with transmembrane transport and oxidation–reduction activities responsible for non-target site resistance in *Poa annua*.	Laforest et al., 2021
Metabolomics	Clodinafop–propargyl	*Avena fatua*	Metabolic fingerprinting analysis was used to examine the changes in the metabolome of *Avena fatua* L. exposed to a gradient of the recommended dose of clodinafop-propargyl, which shows that even a 10,000-fold dilution of the recommended dose could induce a significant change in the plant’s metabolism and this change is permanent over the biological cycle	Tafoya-Razo et al., 2019
			Metabolic fingerprinting has been done using DIESI-MS to determine the metabolic expression of the populations. Researchers found four different metabolic expression patterns.	Tafoya-Razo et al., 2022
	Pinoxaden, mesosulfuron-methyl		The metabolic fingerprint of double herbicide resistant *Avena fatua* showed that the biotype had a markedly different metabolic pattern under control conditions and that this difference was accentuated after herbicide treatment.	Torres-García et al., 2018
	Glufosinate	*Stenotaphrum secundatum*	GC–MS based untargeted metabolomics study has been done to assess the delayed response of glufosinate treatment of transgenic herbicide-resistant buffalo grasses. The authors found significant metabolic alterations in the sensitive wild type, with the up-regulation of several amino acids due to glufosinate-induced senescence.	Boonchaisri et al., 2020
	Glyphosate	*Amaranthus palmeri*	The specificity of metabolic perturbations induced by glyphosate has been investigated on resistant and susceptible *Amaranthus palmeri*. The study found that the phytochemical responses are stress-specific rather than biotype-specific.	Sandhu et al., 2023
			Non-targeted GC–MS and LC–MS metabolomic profiling was conducted to examine the innate physiology and the glyphosate-induced perturbations in sensitive and resistant biotypes. The results found that the lethality associated with an amino acid pool imbalance and accumulation of the metabolites of the shikimate pathway upstream from 5-enolpyruvylshikimate-3-phosphate synthase.	Zulet-Gonzalez et al., 2023
		*Ipomoea lacunosa*	Metabolomic profiling was conducted to examine the innate physiology and the glyphosate induced perturbations in two biotypes of *I. lacunosa* that had contrasting glyphosate tolerance. They found that abundance of transport-sugar and amino acid is playing major role for glyphosate sensitivity.	Maroli et al., 2018b
	Pyraclonil, fentrazamide, benzobicyclon, propyrisulfuron, imazosulfuron	*Schoenoplectus juncoides*	Non-targeted and targeted metabolomics was performed using ALS inhibitor treated *Schoenoplectus juncoides* and they identified internal metabolite markers for ALS inhibition, with excellent selectivity for ALS inhibitors and herbicides with different MOAs in various weed species.	Hikosaka et al., 2021
	Syncarpic acid-3	*Amaranthus tuberculatus*	A LC-MS-based untargeted metabolomics was conducted in *Amaranthus tuberculatus* and authors found that Phase I metabolite, generated by cytochrome P450-mediated alkyl hydroxylation, was detected but was not associated with resistance. A Phase II glutathione–SA3 conjugate was associated with resistance.	Concepcion et al., 2021

It is a well-known fact that, within a biological system, transcript abundance does not always translate to protein abundance due to several biological and technical factors. These factors might include post-transcriptional and translational regulatory factors, mRNA stability, protein degradation and turnover, etc. Additionally, even if a protein is formed, it may not inevitably be functional (Liu et al., 2016; Manavella et al., 2023). In this context, correlating the transcriptome and the proteome might be misleading. Therefore, we strongly advise systematically integrating the transcriptome and metabolome data instead. However, it is important to note that post-translational modifications (such as phosphorylation and acetylation) cannot be identified through transcriptomics and metabolomics alone. In such cases, mounting up more data (from other omics such as proteomics and genomics) can boost our understanding of biological complexities and minimize bias. To date, even though there is a lack of research on using the transcriptome and metabolome data for integrated analysis in herbicide resistance research, a few such studies have been conducted in other plants. For example, in a study conducted by Xu et al. (2023a), the authors combined transcriptome and metabolome data from two contrasting apple species (cold-resistant vs cold-sensitive cultivars) to identify the key metabolic pathways involved in response to cold stress. They identified differentially expressed genes (DEGs) and differentially expressed metabolites (DEMs), which provided novel insights into the mechanisms of cold resistance in apple trees in response to cold stress during dormancy (Xu et al., 2023a). In another study conducted in 2023, the authors used integrated transcriptomic-metabolomic analysis to investigate the possible roles of flavonoids involved in resistance to powdery mildew in wheat. They identified DEGs and differentially accumulated flavonoids and validated them using qRT-PCR and biochemical analyses (flavonoids and malondialdehyde content measurements, antioxidant enzyme activities), respectively (Xu et al., 2023b).

**Figure 1:**
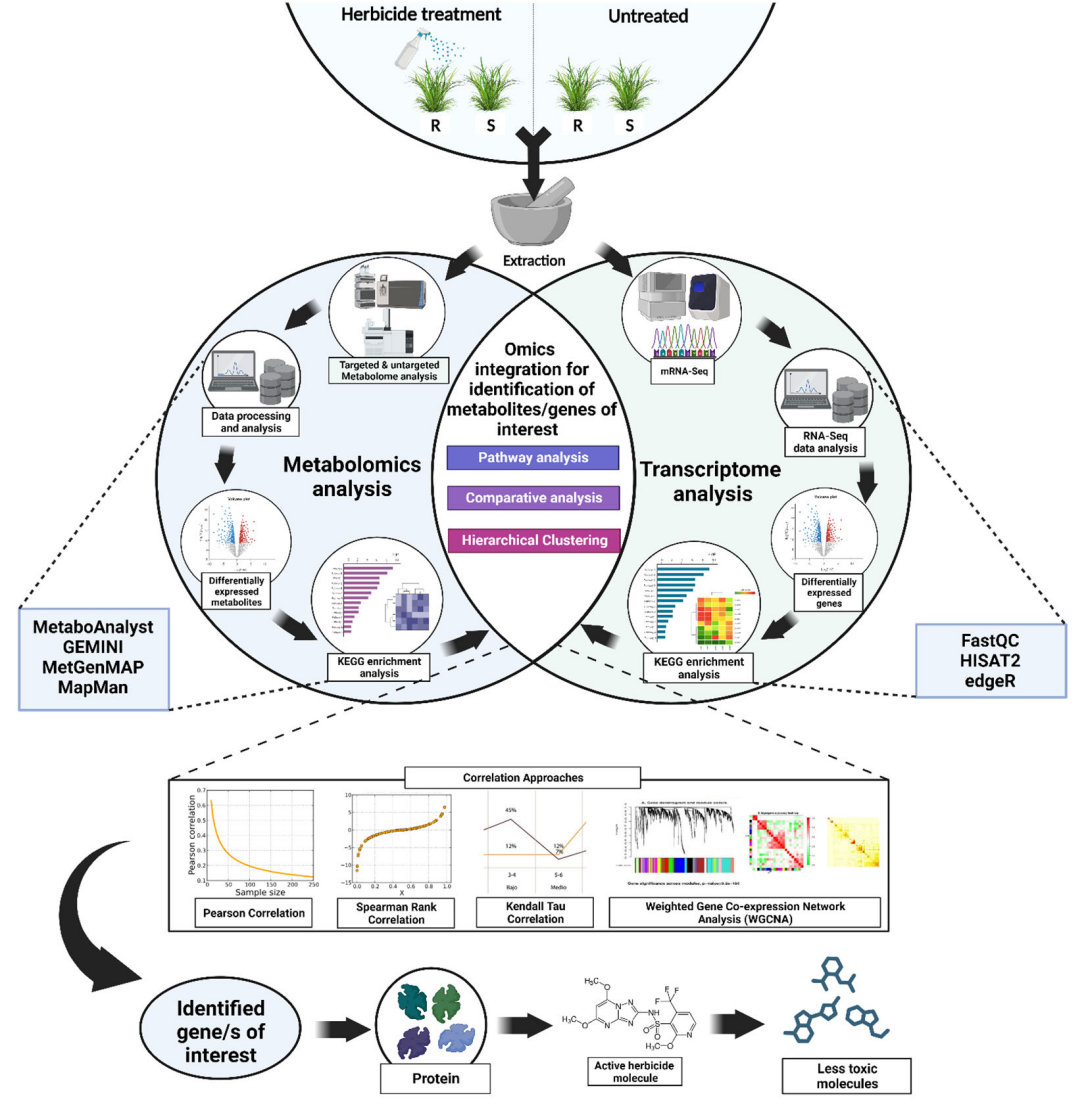
A simplified framework showing the possible experimental design based on integrated transcriptomics and metabolomics in weeds. The RNA-Seq transcriptome analysis will help to identify the differentially expressed genes under herbicide stress (such as genes encoding cytochrome P450s, GSTs, GTs, transporter proteins, oxidases, peroxidases etc.). Thereafter, the metabolite profiling using metabolomics [using Gas Chromatography/Mass Spectrometry (GC/MS) or Liquid Chromatography/Mass Spectrometry (LC/MS)] will further confirm the involvement of these differentially produced metabolic enzymes. Following the analysis of individual omics data, statistical and bioinformatic techniques, such as correlation analysis using Pearson correlation coefficient, Spearman rank correlation (ρ), kendall’s Tau (τ) and weighted gene coexpression network analysis (WGCNA)., can be used to integrate them. Suitable significance thresholds can be set to identify the significant associations between gene expression and metabolite abundance. The integration of these omics approaches can establish a link between phenotypic outcome to genotypic expression and hence can provide a system-level understanding of herbicide resistance mechanisms in weeds.

Similarly, among the herbicide resistance mechanisms, NTSRs are always believed to be a cumulative effect of several gene families (Ghanizadeh and Harrington, 2017). Hence, the transcriptome can only identify the expression of the important genes, which is often inadequate when studying the weeds as a system. Additionally, the relationship between metabolite and transcript levels might go beyond merely the fact that gene expression affects the global metabolite levels. Alternatively, gene expression might also be regulated by the metabolites, indicating an interconnection between the two (Anjali et al., 2023). Apart from the functional prediction of genes and coexpression analyses, integrated transcriptome-metabolome can also be used to analyze the organizational principle of the whole system (such as systems characterization of pathways) (Xue et al., 2021). In the current scenario, correlating the transcriptome and the metabolome data might allow us to discover the key genes and the metabolic pathways associated with the herbicide resistance in the system. Parallel analysis of transcript and metabolite profiling can expose novel gene-to-metabolite networks and hence can realistically screen candidate genes involve in herbicide resistance (for example, exactly which isoforms of Cyp450s or GSTs or GTs) (Fukushima et al., 2009; Nakabayashi and Saito, 2015). Currently, there are quite a few comprehensible gene-coexpression tools that are specifically designed for plant genomics research. Some of them include Arabidopsis Coexpression Tool (ACT), *Arabidopsis thaliana* transcription factor and protein interaction database (ATTED-II), Genevestigator, Co-expression Platform (CoP), PlantPAN 3.0 and GENEVESTIGATOR V4 (Nakabayashi and Saito, 2015; Zogopoulos et al., 2021). Apart from the mentioned comprehensible gene-coexpression tools, there are also several publicly available tools and software platforms that could assist in the parallel analysis of transcriptome and metabolome data. Such tools include MetaboAnalyst (current version: MetaboAnalyst 5.0), GEMINI (Gene Expression and Metabolism Integrated for Network Inference), Plant MetGenMAP, mixOmics, etc. MetaboAnalyst (https://www.metaboanalyst.ca/MetaboAnalyst/home.xhtml) is a user-friendly web-based platform designed exclusively for metabolomics data analysis that offers a wide range of capabilities, from raw MS spectra processing to comprehensive data normalization and integration with other omics data (Pang et al., 2022). Plant MetGenMAP (http://bioinfo.bti.cornell.edu/cgi-bin/MetGenMAP/home.cgi) is a web-based system that allows the user to identify the changed pathways from gene expression and/or metabolite profile data. This system enables data visualization in a biochemical pathway context (Joung et al., 2009). GEMINI is a software tool designed to infer regulatory networks that connect genes and metabolites, and hence can be considered as an important tool for the integrated analysis of transcriptomics and metabolomics data (Chandrasekaran, 2019). mixOmics is a collaborative R package-based project (between Australia, France, and Canada) that focuses on multi-omics data integration. This tool can be used for integrative analysis of transcriptomics and metabolomics data, with a special emphasis on dimension reduction (Rohart et al., 2017). **Figure 1** describes a schematic framework of the experimental design based on integrated transcriptomics and metabolomics in weeds. Following the analysis of individual transcriptome and metabolome data, statistical and bioinformatic techniques, such as correlation analysis, can be used to integrate them. The coexpression of genes and the production of metabolites can be measured by different types of correlation coefficients such as Pearson correlation coefficient (r), Spearman rank correlation (ρ), Kendall’s Tau (τ) and weighted gene coexpression network analysis (WGCNA). For example, in a study conducted by Yang et al., the authors had used Pearson correlation coefficients and their corresponding *p*-values to discover metabolites and related genes from a combined metabolomic and transcriptomic analysis (Yang et al., 2022b). In another study conducted by Zhang et al., the authors had successfully identified key structural genes responsible for anthocyanin biosynthesis mechanisms in *Vaccinium corymbosum* L. using integrated transcriptome and metabolome analysis. They had also used Pearson correlation coefficient values for screening out the genes of interest (Zhang et al., 2022b). In the context of herbicide resistance mechanism discovery, these tools can be used to assess how changes in gene expression levels correspond to variations in metabolite abundances, and vice versa. Based on the integration results, the genes showing significant correlations with specific metabolites can be identified. Prior knowledge of metabolic pathways in combination with the metabolome result can be used to assess the possible biological relevance of these identified genes to herbicide resistance.

## 4 Summary and outlook: current challenges and way forward

Individually, both metabolomics and transcriptomics are powerful tools for understanding the molecular basis of herbicide resistance. However, determining whether expression of a particular gene or increased concentration of a particular metabolite is the cause or consequence of herbicide adaptation is a difficult task. Such questions can be answered by integrated transcriptome-metabolome studies. Despite significant advances in omics-based studies of herbicide resistance, to the best of our knowledge, to date there have been inadequate efforts made to investigate the power and potential of transcriptome-metabolome correlation analysis in herbicide resistance. This can likely be attributed to the limited availability of genomic and metabolome resources in weeds. Even though RNA-seq studies have been successfully used to study evolutionary processes in weeds, they have their own challenges. The main issue to address is the proportion of mismatches, especially when genetic differences between species are high. In such cases, the portion of the reads mapping ambiguously to more than one contig increases, which might lead to erroneous result interpretation and further downstream processes (Fukushima et al., 2009). Hence, high-resolution genomic analyses of weedy plants will be needed in the future. Alongside transcriptome studies, metabolome studies on weeds also have numerous challenges, which might range from constrained genomic resources and reference databases to the complexity of the metabolite profiles. In weeds, the lack of reference databases can make it difficult to identify and annotate all the metabolites. Furthermore, there might be many anonymous metabolites, which can also impede precise result interpretations and hence make biomarker discovery difficult in weeds.

Besides individual omics, the application of integrated omics approaches to weeds can also be challenging and requires careful consideration. One of the most important data integration challenges is that the transcriptome and metabolite information do not correlate for some of the genes and metabolites. This might be due to complicated regulatory networks ranging from feedback loops to co-regulation and compensatory mechanisms (Stelling et al., 2002; Vemuri and Aristidou, 2005). Additionally, metabolites within cells can have varying turnover rates. Hence, even if gene expression remains constant, that does not imply that metabolite levels will also remain constant (Li et al., 2022). Hence, in these cases, gene-metabolite correlations may not be evident from short time scale experiments. Also, there can be some technical factors and threshold effects involved, which can initiate weak correlations. For example, minor changes in gene expression might not lead to measurable changes in metabolite levels until a certain threshold is reached, or there might be some abnormalities regarding the normalization methods and data preprocessing choices that might obscure the true correlations. Addressing these challenges might require sophisticated and refined statistical analysis methods, and weeds might have unknown statistical challenges that need to be addressed (Kumar et al., 2017). Another challenge might be the huge diversity among the weed species, resulting in substantial physiological and genetic heterogeneity even within a single species. Due to species diversity, each might have its own unique genetic makeup and biological characteristics, and hence the resistance mechanisms might also vary significantly (Owen, 2016). High species diversity might also limit the broader applicability of research findings from multi-omics data since the perceptions gained from one environment may not apply directly to another. This scenario might demand more advanced bioinformatics pipelines to accommodate the unique genomic features of each species. This will require specialized expertise. However, the most crucial challenge in integrated transcriptomic-metabolomic analyses is based on the fact that there is no direct connection between metabolite and transcript. The differentially expressed transcripts cannot be mapped to a single metabolome database. Hence, the data analysis becomes more complicated; however, this impasse can be overcome by reducing the number of metabolites. Comparative metabolomic analyses, such as comparison between herbicide-resistant and susceptible weeds and identification of the specific features, can be implied to reduce the undesirable metabolites. Nevertheless, despite the challenges, transcriptomic-metabolomic integration is a powerful combination and a fascinating field of research that undeniably requires additional exploration and the involvement of more geneticists and weed scientists.

## Author contributions

MKS: Conceptualization, Data curation, Formal analysis, Investigation, Supervision, Writing – original draft, Writing – review & editing. SB: Data curation, Writing – original draft, Writing – review & editing. RB: Data curation, Formal analysis, Investigation, Software, Writing – original draft, Writing – review & editing. KH: Formal analysis, Supervision, Validation, Writing – review & editing. JS: Formal analysis, Funding acquisition, Project administration, Resources, Supervision, Validation, Writing – review & editing.
